# PSMA Ligand Conjugated PCL-PEG Polymeric Micelles Targeted to Prostate Cancer Cells

**DOI:** 10.1371/journal.pone.0112200

**Published:** 2014-11-11

**Authors:** Jian Jin, Bowen Sui, Jingxin Gou, Jingshuo Liu, Xing Tang, Hui Xu, Yu Zhang, Xiangqun Jin

**Affiliations:** 1 Department of Pharmaceutics, College of Pharmacy Sciences, Jilin University, Changchun, People's Republic of China; 2 Department of Pharmaceutics, Shenyang Pharmaceutical University, Shenyang, People's Republic of China; University of Illinois, United States of America

## Abstract

In this content, a small molecular ligand of prostate specific membrane antigen (SMLP) conjugated poly (caprolactone) (PCL)-b-poly (ethylene glycol) (PEG) copolymers with different block lengths were synthesized to construct a satisfactory drug delivery system. Four different docetaxel-loaded polymeric micelles (DTX-PMs) were prepared by dialysis with particle sizes less than 60 nm as characterized by dynamic light scattering (DLS) and transmission electron microscope (TEM). Optimization of the prepared micelles was conducted based on short-term stability and drug-loading content. The results showed that optimized systems were able to remain stable over 7 days. Compared with Taxotere, DTX-PMs with the same ratio of hydrophilic/hydrophobic chain length displayed similar sustained release behaviors. The cytotoxicity of the optimized targeted DTX-PCL_12K_-PEG_5K_-SMLP micelles (DTX-PMs2) and non-targeted DTX-PCL_12K_-mPEG_5K_ micelles (DTX-PMs1) were evaluated by MTT assays using prostate specific membrane antigen (PSMA) positive prostate adenocarcinoma cells (LNCaP). The results showed that the targeted micelles had a much lower IC50 than their non-targeted counterparts (48 h: 0.87±0.27 vs 13.48±1.03 µg/ml; 72 h: 0.02±0.008 vs 1.35±0.54 µg/ml). *In vitro* cellular uptake of PMs2 showed 5-fold higher fluorescence intensity than that of PMs1 after 4 h incubation. According to these results, the novel nano-sized drug delivery system based on DTX-PCL-PEG-SMLP offers great promise for the treatment of prostatic cancer.

## Introduction

Polymeric micelles have received considerable attention as promising anticancer drug carriers because of their remarkable advantages, such as small size, narrow size distribution, high biocompatibility, and solubilization of hydrophobic drugs [1], [2], [3], [4], [5]. Self-assembled polymeric micelles with core/shell structures enable the system to incorporate poorly water-soluble drugs in the hydrophobic core and protect them from degradation in physiological media [6]. For example, the hydrophobic core of the micelles composed of PCL-PEG offers a reservoir for the incorporation of drugs, while the pegylated shell along with its nanoscopic size guarantees the carrier remain un-recognized by the reticuloendothelial system and undergo a long-circulation period in the blood [7], [8], [9].

Although polymeric micelles exhibited a number of advantages, one major challenge is their site-specific drug delivery. Ligand-modified polymeric micelle drug delivery systems are capable of site-specific drug delivery. Recently, numerous active targeting delivery systems have been designed by conjugating NPs with ligands that bind specifically to the biomarkers of extracellular domains of cancer cells. PSMA as folate hydrolase I and glutamate carboxypeptidase II, is a well-known transmembrane protein [10] over expressed on prostate cancer epithelial cells [11], [12] and has been shown to have great potential for prostatic cancer (PCa) therapy. PSMA has a low expression in normal prostate epithelial cells and benign prostatic hyperplasia. It is also expressed in the neovasculature of most other solid tumors but not in the vasculature of normal tissues [13], [14]. All of these characteristics make PSMA an attractive biomarker for the detection, diagnosis, and treatment of PCa [15], [16]. A novel small molecular ligand ((S)-2-(3-((S)-5-amino-1-carboxypentyl) ureido) pentanedioic acid, SMLP) binding specifically to PSMA has demonstrated its potential in the treatment of cancer in recent years [17]. The urea-based PSMA inhibitor, SMLP, has a high affinity for PSMA due to strong hydrogen bonding [10]. Hrkach and Langer *et al.* developed ACUPA (PSMA ligand) conjugated DTX NPs composed of PEG-b-PLGA or PEG-b-PLA using a nano-emulsification method to target PSMA and evaluated the anti-tumor efficacy of the NPs *in vitro* and *in vivo* [18]. The excellent potential offered by vehicle-ligand targeting PSMA suggests the necessity in developing more diversified preparation processes and carrier-materials in this field.

**Figure 1:**
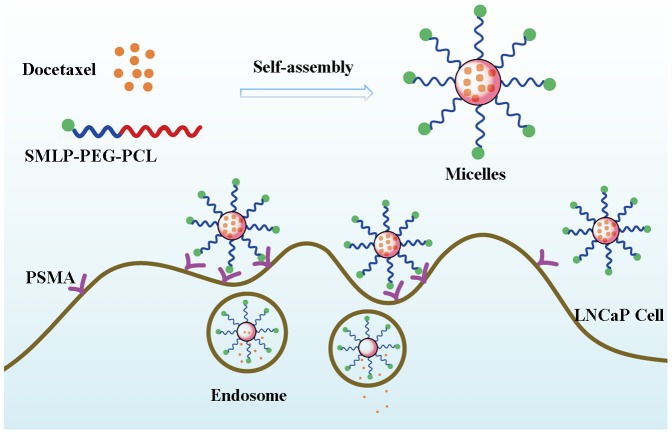
Schematic illustration of DTX-PCL-PEG-SMLP micelles targeted to PSMA.

In this study,a nano-sized self-assembled drug delivery system based on ligand-conjugated PEG-b-PCL micelles was found to show great promise in the field of targeted drug delivery. Copolymers of PCL and PEG are both well-known biodegradable and biocompatible materials widely used in biomedical field [19], [20], [21], [22], [23]. Due to the introduction of glycolic acid (GA) and lactic acid (LA), which disrupted the ordered structure of the molecular chains, PLGA showed low crystallinity. As a result, micelles with cores of PCL which showed higher crystallinity are more stable than those with PLGA cores. Moreover, because PLGA is a random copolymer, it is relatively difficult to control the ratio of GA to LA precisely in large-scale production. However, the ratio of the two monomers is a key factor to influence the property of PLGA [24]. So PCL was used as the core-forming block due to its better stability and ease to produce. PCL-mPEG or PCL-PEG-COOH was synthesized by ring-opening polymerization of ε-caprolactone initiated by mPEG-OH or HOOC-PEG-OH [25], [26]. PCL-mPEG and PCL-PEG-SMLP micelles were prepared using DTX as a model drug to examine the cytotoxic effects on LNCaP cells. Also, a schematic illustration of preparation and endocytosis process of DTX-PCL-PEG-SMLP is shown in Figure 1.

## Materials and Methods

### Materials

L-glutamic acid di-tertbutyl ester hydrochloride and H-Lys(Z)-Ot-Bu hydrochloride were obtained from En lai Biological Technology Co., LTD (Chengdu, People's Republic of China). mPEG-OH (Mw: 2 kDa) and mPEG-OH (Mw: 5 kDa) (Aladdin Agent Co., Shanghai, P R China) and OH-PEG-COOH(Mw: 2 kDa),OH-PEG-COOH(Mw: 5 kDa) (Shanghai Seebio Biological Technology Co., shanghai, P R China) were dehydrated by azeotropic distillation with toluene before use. DTX (Shanghai Sanwei Pharma Ltd, Co, Shanghai, P R China) and the cellulose ester dialysis bag with a molecular cut-off of 7000 Da (Bioscience Ltd, Co, Shanghai, P R China) were used as received. ε-Caprolactone (ε-CL, Aladdin Agent Co., Shanghai, P R China) was dried over CaH_2_ at room temperature for 48 h and distilled under reduced pressure. Stannous octoate was purchased from Aladdin Agent Co (Shanghai, P R China). All organic solvents used in the synthesis procedures were purchased from the National Medicine Chemical Reagent Ltd Co (Shanghai, P R China).

The prostate LNCaP and PC3 cell lines were obtained from the Type Culture Collection of the Chinese Academy of Sciences (Shanghai, China). LNCaP Cells were cultured using cell-bind culture bottles (Corning, USA).

### Methods

#### Synthesis of PCL-mPEG (PMs1) and PCL-PEG-SMLP (PMs2) copolymers

Copolymers with a range of block lengths were prepared by ring-opening copolymerization of ε-CL initiated by hydroxyl of PEG. Briefly, a predetermined amount of ε-CL and stannous octoate were added to a reaction vessel containing mPEG-OH or OH-PEG-COOH under a dry argon atmosphere (stannous octoate/ε-CL in 1∶1000 molar ratio). Then, the reaction vessel was placed in an oil bath and maintained at 120°C for 24 h. Then the crude copolymers were dissolved in DCM and precipitated in cold diethyl ether to remove the un-reacted monomer and oligomer. Then, the product was filtered and dried to obtain a white precipitate.

PCL_12k_-PEG_5k_-COOH (1 g, 0.059 mmol) was dissolved in 5 ml anhydrous tetrahydrofuran (THF) with 1-ethyl-3-[3-dimethylaminopropyl]-carbodiimide hydrochloride (EDC) (57.4 mg, 0.3 mmol, 5 equiv) and N-hydroxysuccinimide (NHS) (27.6 mg, 0.24 mmol, 4 equiv). Then, the solution mixture was stirred at room temperature for over 12 h under argon atmosphere. The PCL_12k_-PEG_5k_-NHS copolymer was precipitated in ice-cold diethyl ether to afford a white precipitate which was collected and dried to obtain the desired product as a white powder (yield, 90%). SMLP (300 mg) was dissolved in anhydrous THF (20 ml) to prepare 10 mg/ml (SMLP/THF) aqua. PCL-PEG-NHS (500 mg, 0.03 mmol) and diisopropylethylamine (0.7 ml) were added to 5 ml (SMLP/THF) aqua, and the reaction solution was stirred at room temperature for 20 h under argon. After completion of the reaction, the solution was purified by dialysis for 24 h and dried by lyophilization to obtain a white flocculent powder (yield 90%). The structure of final copolymer was characterized by ^1^H NMR spectroscopy.

PCL_4.8K_-mPEG_2K_ and PCL_4.8k_-PEG_2k_-SMLP were prepared using the same method previously stated.

#### Polymer characterization

The ^1^H-nuclear magnetic resonance (^1^H NMR) spectra of all samples were recorded on a Bruker DMX 300 or 600 spectrometer (Billerica, MA). Chemical shifts (δ) were given in ppm using tetramethylsilane as the internal standard. Fourier transform infrared spectroscopy spectra were recorded on a Bruker Tensor 27 spectrometer, and samples were prepared using KBr disks (Scharlau Chemie, Barcelona, Spain). Gel permeation chromatography (GPC) assay was performed on a Waters 1515 GPC instrument (Waters Corp, Milford, MA) equipped with three styragel columns (Waters Corp; 10^5^, 10^4^, and 103 Å) in tandem and a 2414 differential refractive index detector. DMF was selected as the eluent at a flow rate of 1.0 ml/min at 35°C. The sample concentrations were approximately 2 mg/ml. The molecular weights were calibrated using polystyrene standards.

#### Preparation of polymeric micelles

DTX-loaded micelles were prepared by dialysis. First, 7 mg DTX and 50 mg copolymer were completely dissolved in 2 ml THF. Then 4 ml phosphate-buffered saline (PBS; 10 mM, pH 7.4 or 10 mM, pH 5.5) was added drop-wise to the solution under continuous stirring for one hour. Then, THF was removed by dialysis against PBS (10 mM, pH 7.4 or 10 mM, pH 5.5) over 24 h using a cellulose ester dialysis bag (MWCO: 7000 Da). The outer medium was replaced three times (2, 6, and 12 hours). Finally, the mixture was passed through a 0.45 µm filter membrane to remove any precipitants.

#### Drug-loading content and encapsulation efficiency

To determine the drug-loading content and encapsulation efficiency, 500 µl DTX-loaded micellar solution and 5 ml THF were transferred to a 25 ml volumetric flask, sonicated at 180 W for 10 minutes in an ultrasonic bath, and then diluted with mobile phase. The concentration in the resulting solution was then determined by HPLC. Chromatographic analysis was performed using a Hitachi L-2130 pump and a Hitachi L-2400 UV-Vis detector operated at a wavelength of 230 nm, using a Unitary C18 column (5 µm, 150×4.6 mm). A mobile phase of acetonitrile and water (60/40, v/v) was selected. The flow rate was set at 1 ml/min. The peak area response versus the DTX concentration was linear over the range of 0.5–30 µg/ml (r^2^ = 0.9999).

The drug-loading content and encapsulation efficiency were calculated from the following equations: 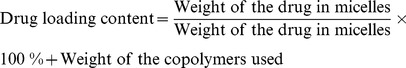


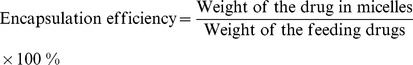



#### Particle size measurements

The particle size and distribution of micelles were measured by DLS using NICOMP 380 Submicron Particle Sizer (Particle Sizing Systems, Santa Barbara, CA). A laser beam at a wavelength of 632.8 nm was used. The scattering angle was set at 90° when measurements were conducted.

#### Surface morphology

Samples for TEM observation were prepared by placing a drop of sample solution (2 mg/ml for copolymer) on to a copper grid coated with carbon. Excess solution was wiped away with filter paper. The grid was allowed to dry for a further 15 minutes. Then, the samples were examined using a Hitachi H-600 TEM operated at an accelerating voltage of 100 kV.

#### *In Vitro* Release

The *in vitro* DTX release kinetics of drug-loaded micellar solutions or DTX injection (Taxotere, Sanofi-Aventis, Paris, France) containing 300 µg DTX were performed by dialysis diffusion. The drug-loaded micellar solution and free drug solution were placed in the dialysis bags (MWCO: 14000). These bags were immersed in 15 ml PBS pH 7.4 (10 mM) or pH 5.5 (10 mM) containing 0.5% w/v Tween 80. Subsequently, the bottles were placed in a shaking incubator at a shaking speed of 100 rpm under 37°C±0.5°C. All release media were replaced with fresh PBS at predetermined intervals (1 h, 2 h, 4 h, 8 h, 12 h, 24 h, 36 h, 48 h, 60 h, 72 h, 84 h and 96 h) in order to measure the drug concentration. The concentration of DTX was measured by HPLC.

#### Cell Culture and Cytotoxicity

The prostate LNCaP and PC3 cell lines were cultured in RPMI 1640 medium and Ham's F12K (Invitrogen, USA), supplemented with 10% fetal bovine serum (Hyclone, USA), respectively. The cultures were maintained in a 95% air humidified atmosphere containing 5% CO_2_ at 37°C.

MTT assay was conducted to evaluate the cytotoxicity of DTX-PMs1 (nontargeted) and DTX-PMs2 (targeted). LNCaP cells were suspended in culture medium and seeded at 5000 cells/well in 96-well plates for 24 h. Then, dispersed DTX-PMs1, DTX-PMs2, and the DMSO solution of DTX (DSD) containing four drug concentrations (0.1, 1, 10 and 20 µg/ml) in each sample were incubated in LNCaP cells. Finally, the cell viability was determined after 48 h and 72 h using a Microplate Reader (Bio-Rad imark, USA).

The IC50 for each system was then calculated. All assays were conducted with five parallel samples.

#### Cellular uptake studies

In this study, coumarin 6 was used as a fluorescence probe. Androgen-dependent and androgen-independent prostate cell lines (LNCaP and PC3, respectively) were used. Cellular uptake of targeted and non-targeted PMs (200 µg/ml) carrying coumarin 6 (100 µg/ml) (PMs1 and PMs2, respectively) were conducted on LNCaP and PC3 cell lines to investigate the influence of SMLP conjugation on cellular uptake. The cells were incubated in 96-well plates with micelles for 4 h, washed with cold PBS three times, and then fixed with 70% ethanol for 2 h at −20°C. A competitive inhibition study was also conducted using free SMLP to verify whether the PMs were transported into cells in a SMLP-mediated manner. Free SMLP with three different concentrations (4 µg/ml, 20 µg/ml and 100 µg/ml) was added into the medium together with PMs2, incubated for 4 h, washed thrice with cold PBS and fixed with 70% ethanol for 2 h at −20°C. Cell nuclei were stained with Hoechst 33342.

The cells were examined using an ImageXpress Micro XL Widefield High Content Screening System (ImageXpress Micro XL, Molecular Devices, USA) with MetaXpress Software. The images of the cells were determined by the differential interference contrast channel technique and the images of coumarin 6-loaded PMs and the nuclei of the cells stained by Hoechst 33342 were recorded with the following channels: blue channel (Hoechst 33342) with excitation at 350 nm and green channel (coumarin 6) with excitation at 485 nm. Then, MetaXpress Software was used to quantify the fluorescence intensity per cell.

#### Statistics

All data were processed using Origin 8.5 software and presented as mean ± SD, and analyzed using Student's *t*-test. Statistical analyses were performed and P<0.01 was considered as the level of statistical significance.

## Results and Discussion

### Synthesis and characterization of PCL-mPEG and PCL-PEG-COOH copolymers

An amphiphilic block copolymer composed of a PCL block as the hydrophobic part and a PEG block as the hydrophilic part was synthesized via ring-opening polymerization using hydroxyl-terminated PEG as a macromolecular initiator.

**Figure 2:**
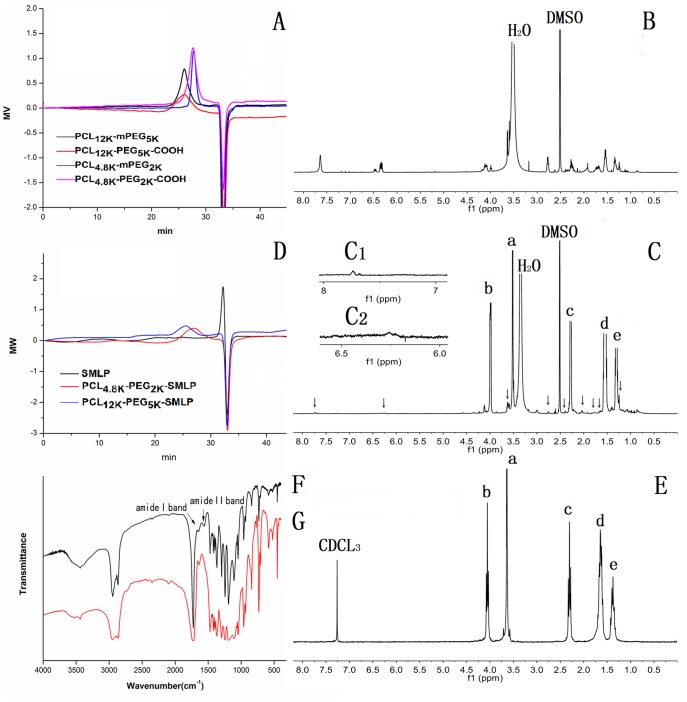
The gel permeation chromatography graphs of four copolymers (A) and SMLP,PCL_4.8K_-PEG_2K_-SMLP and PCL_12K_-PEG_5K_-SMLP (D), the representative ^1^H NMR spectra of SMLP (B), copolymers PCL_12K_-PEG_5K_-SMLP (C) containing SMLP (black arrows) and PCL_12K_-PEG_5K_-COOH (E) with the peaks of PCL_12k_-PEG_5k_ segment (a-e) and Infrared spectra graph of PCL_12k_-PEG_5k_-SMLP (F) and PCL_12K_-PEG_5K_-COOH (G) copolymers.

The molecular weights of the copolymers were calculated from the ^1^H NMR data by comparing the peak intensities of the methylene protons of PEG with the methylene protons of PCL, as shown in Figure 2E. The ratios of the hydrophobic block to the hydrophilic block were determined from the relative intensities of the PCL proton signal at 2.31 ppm and the PEG proton signal at 3.62 ppm. For GPC analysis, only one peak appeared in the GPC curve (Figure 2A), which means that the ring-opening copolymerization of ε-caprolactone with PEG-OH was complete and all the residues were removed after purification. The polydispersity of the copolymer (Mw/Mn) is outlined in Table 1.

**Table 1:** Characterization data of block copolymers and DTX-PMs.

Polymer	Feed ratio^a^ (feed DP)	Final DP^b^	PDI^c^	DTX-PMs	Mean Diameter (nm)	PDI	DLC (%)	EE (%)
**PCL_12K_-mPEG_5k_**	110	104	1.08	**PCL_12K_-mPEG_5k_**	51.4±1.3	0.038±0.04	8.2±0.3	64.2±2.9
**PCL_12K_-PEG_5K_-COOH**	110	106	1.14	**PCL_12K_-PEG_5K_-COOH**	50.5±1.1	0.044±0.06	8.4±0.2	65.7±1.4
**PCL_4.8_K-PEG_2K_**	44	42	1.03	**PCL_4.8K_-PEG_2K_**	37.1±0.5	0.038±0.03	7.3±0.3	56.4±2.6
**PCL_4.8K_-PEG_2K_-COOH**	44	42	1.07	**PCL_4.8K_-PEG_2K_-COOH**	38.6±0.7	0.033±0.02	7.5±0.4	58.0±3.4

(n = 3).

**Notes**: ^a^Calculation of feed ratio by nPCL/nPEG. ^b^Degree of polymerization (DP) determined by ^1^H NMR. ^c^Molecular weight polydispersity index (PDI) determined by GPC (Mw/Mn).

**Abbreviations**: Drug-loading content, DLC; Encapsulation efficiency, EE.

### Synthesis and characterization of PCL-PEG-SMLP

Surface functionalization of the copolymer PCL-PEG-COOH with SMLP was achieved under standard amide coupling conditions in the presence of EDC and NHS [27], [28]. The coupling efficiency with amine nucleophiles can be increased by the formation of an NHS ester intermediate [29].

**Figure 3:**
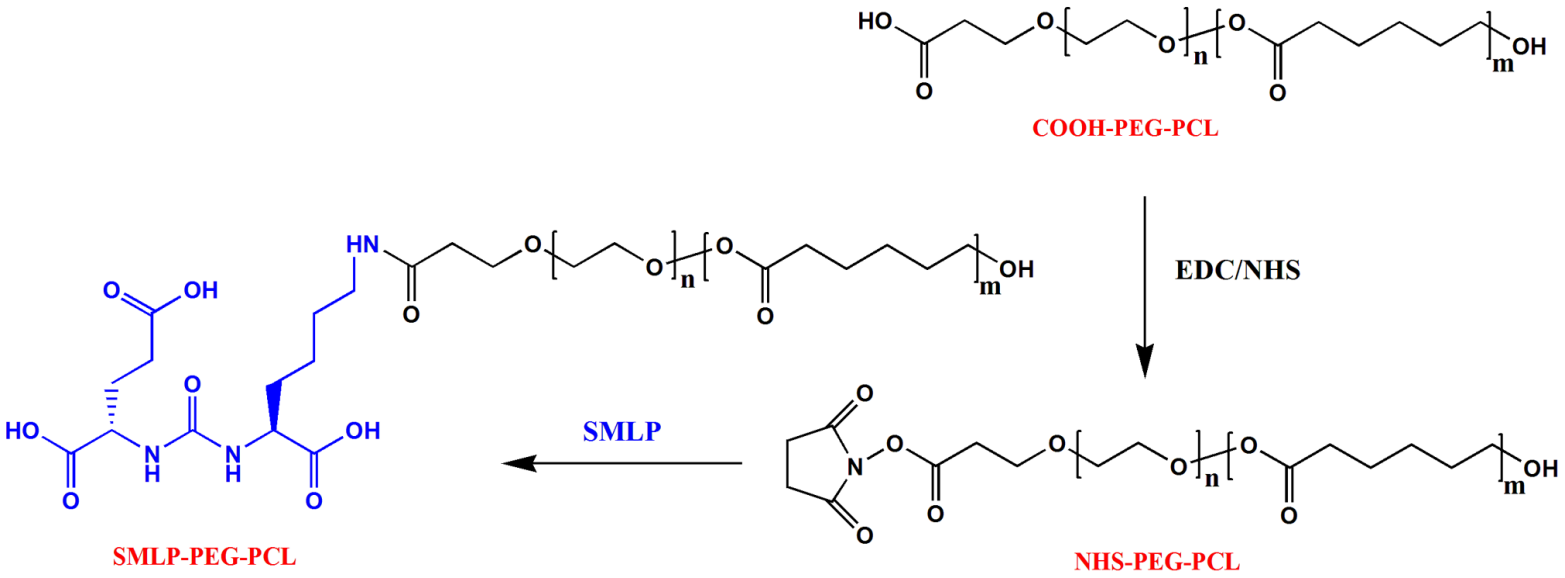
Synthetic representation of the chemical reaction for preparation of PCL-PEG-SMLP copolymer.

The synthesis of polymer PCL–PEG–SMLP was accomplished by the following method. First, the carboxyl of PCL-PEG-COOH was activated with EDC and NHS to achieve the intermediate PCL-PEG-NHS. Then, the active ester (NHS) of PCL-PEG-NHS was reacted with the amine functional group of the SMLP to obtain the final polymer PCL-PEG-SMLP (Figure 3).

The polymer of PCL_12k_–PEG_5k_–SMLP was characterized using FT-IR, as depicted in Figure 2F. The salient peaks shown in Figure 2F at 1671 and 1557 cm^−1^ were attributed to amide band I (carbonyl group) and amide band II (amino group) respectively, while the disappearance of these peaks (Figure 2G) indicated the formation of the amide bond between SMLP and PCL_12k_-PEG_5k_-COOH. The structure of the conjugate was further examined by ^1^H NMR. Figure 2C shows the ^1^H NMR spectrum of the conjugation of the ligand and copolymer PCL_12k_-PEG_5k_-COOH. The characteristic signal appearing at 3.60 ppm (a) was assigned to the PEG unit. The peaks of the PCL units appear at 4.04–4.08 ppm (b), 2.28–2.33 ppm (c), 1.61–1.70 ppm (d) and 1.35–1.43 ppm (e), as shown in Figure 2C. Moreover, the signals at 2.49 ppm and 3.25–3.49 ppm were assigned to the solvent peak (DMSO) and water peak, respectively. According to Figure 2E, there are no SMLP-related signals shown in the ^1^H NMR spectrum of unconjugated PCL_12k_-PEG_5k_-COOH, indicating that there is no interference with SMLP signals shown in Figure 2C.Comparing the peaks of PCL-PEG-SMLP in Figure 2C and the peaks of ligand SMLP in Figure 2B, the chemical shifts were identical. Combing the results of FT-IR and ^1^H NMR showed that the ligand SMLP had been successfully conjugated to PCL_12K_-PEG_5K_-COOH. The purity of ligand conjugated polymers, which is critically related to the *in vitro* performance of the micelles, was verified by gel permeation chromatography. As shown in Figure 2D, no trace of free SMLP was observed in the chromatograms of either PCL_12K_-PEG_5K_-SMLP or PCL_4.8K_-PEG_2K_-SMLP, indicating that the excessive free ligand was completely removed.

### Preparation and characterization of micelles

In this work, dialysis was employed to prepare the docetaxel-micelles, leading to the successful preparation of nontargeted [PCL_12K_-mPEG_5k_ (PMs1), PCL_4.8k_-mPEG_2K_ (PMs3)] and targeted [PCL_12K_-PEG_5K_-SMLP (PMs2), PCL_4.8K_-PEG_2K_-SMLP (PMs4)] micelles. In addition, nanoparticles with diameters larger than 100 nm are more likely to be eliminated by the reticuloendothelial system [30], while their counterparts with diameters less than 100 nm were more likely to accumulate in tumor tissues [31], [32].

**Figure 4:**
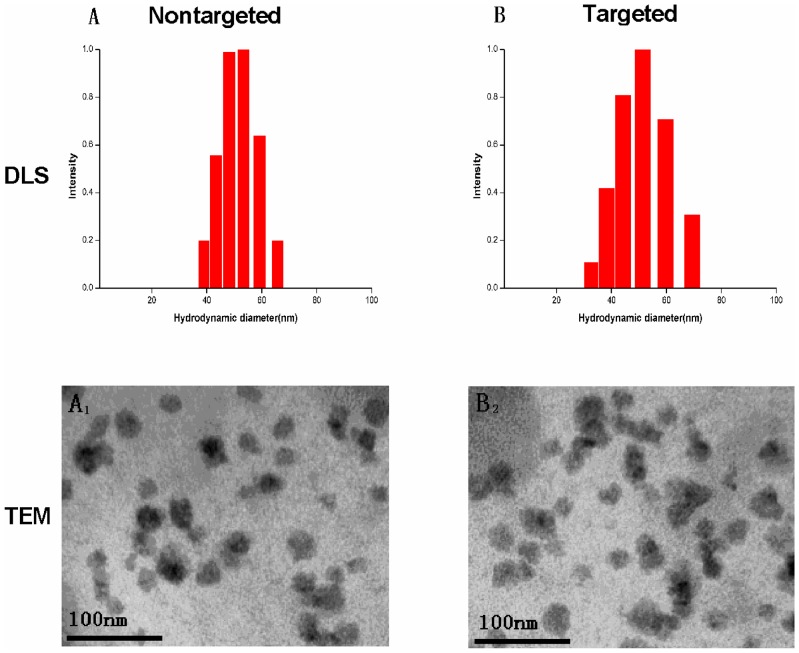
The representative DLS graphs of the DTX-PMs1 (A), and DTX-PMs2 (B), respectively. TEM graphs of DTX-PMs1 (A_1_) and DTX-PMs2 (B_2_).

The average diameters of micelles (PMs1, PMs2, PMs3 and PMs4) prepared by dialysis were 51.4±1.3 nm, 50.5±1.1 nm, 37.1±0.5 nm and 38.6±0.7 nm, respectively. The polydispersity index (PDI) values of the four micelles are shown in Table 1. The DLS graphs of PMs1 and PMs2 are shown in Figure 4 (A, B). The morphology and low PDI of the micelles were further confirmed by TEM imaging. The TEM photograph (Figure 4, A_1_ and B2) of PMs1 and PMs2 were in accordance to the results of DLS. The smaller diameters of the PMs obtained from the TEM tests compared with DLS could be ascribed to the shrinkage of the PEG shell induced by water evaporation before TEM measurement [33]. As a result, the diameter given by DLS was bigger than that of TEM due to the hydration of the PEG shell.

**Figure 5:**
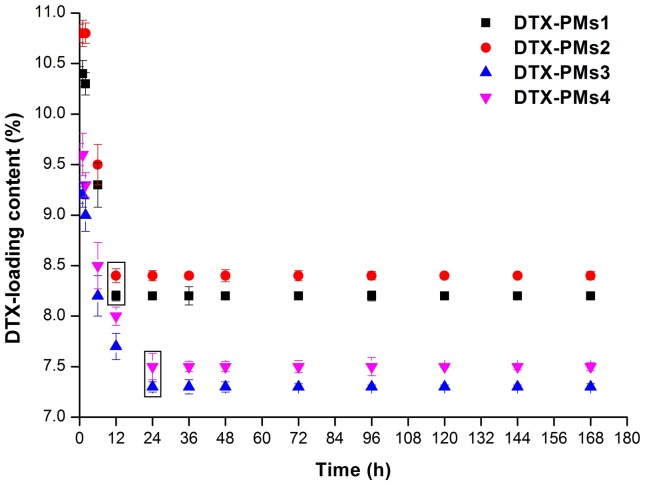
The short-term stability of the DLC of four DTX-PMs stored at room temperature for 7 days. (n = 3).

To evaluate the maximum drug-loading content and drug-loading efficiency of the four micelles, a simple short-term stability study of the DTX-loaded content was performed and the results are shown in Figure 5. First, excess DTX was added during the preparation of the four DTX-PMs. The over-loaded PMs were kept at room temperature and sampled at predetermined time. Then, the DTX-loading content of the samples was measured by HPLC using the method described. The profile showed that the initial DTX-loading content of the four PMs was 10.4%, 10.8%, 9.6% and 9.8%, respectively. The values of DTX-loading content fell gradually and remained constant after 12 h for PCL_12k_-PEG_5k_ and 24 h for PCL_4.8k_-PEG_2k_ (Figure 5, circled in squares). The reduction in drug-loading content may be due to the occurrence of phase separation between DTX and PCL. The drug-loading content after a 7 days test period could be deemed as the capacity of PCL for loading DTX. Also, the higher capacity of PMs1 and PMs2 compared with PMs3 and PMs4 could be ascribed to the longer PCL chains of PMs1 and PMs2. With the same ratio of the hydrophilic block length to the hydrophobic block length, the final drug-loading content and encapsulation efficiency of the four PMs are shown in Table 1. These results showed the a good short-term stability of the DTX-loading content, the drug-loading content and efficiency, confirming that the micelles based on PCL_12K_-PEG_5K_-SMLP and PCL_12K_-mPEG_5k_ copolymers were optimal formulations.

### *In vitro* release

**Figure 6:**
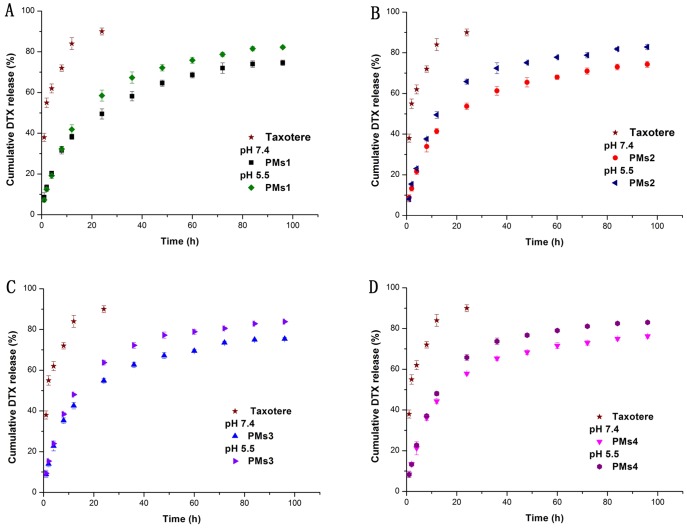
*In vitro* release profile of DTX from the four types of PMs solution at 37°C in PBS (10 mM, pH 7.4) or PBS(10 mM,pH 5.5), in comparison with Taxotere. (n = 3).

The *in vitro* release behavior of four DTX-PMs was investigated by the dialysis diffusion method [34]. The release behavior of Taxotere was used as a control, and the DTX release profiles of the four PMs at pH 7.4 (simulated environment of normal tissues) and pH 5.5 (simulated environment of tumor tissues) are shown in Figure 6. Almost 90% of the DTX was released from Taxotere within 24 h. Unlike Taxotere, all micelles exhibited a fast release of DTX at the initial stage (first 24 h) and a sustained release over the following 72 h. Moreover, the similar release profiles of PMs1 and PMs2 indicated that ligand conjugation did not influence the release pattern.

For the four PMs, because the poly ester structure of PCL is sensitive to acid, the release of DTX from micelles was slower at pH 7.4 than that at pH 5.5. This effect promoted the release of DTX in tumor tissues and in endosomes which are more acidic than blood [35]. The amount of non-released DTX was 20–25%. This proportion of drugs existed in the micellar cores; trapped in precipitations generated by heat and tween 80 induced micellar breakdown and adsorbed on dialysis bag and glassware [36].

### Cell cytotoxicity

**Figure 7:**
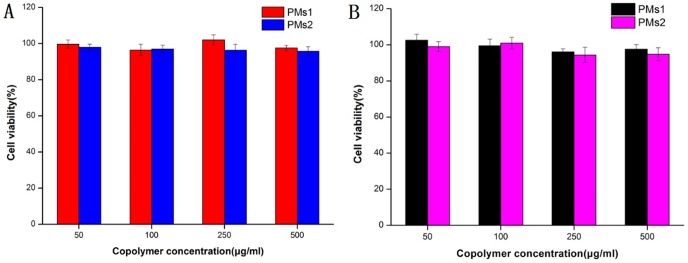
Different concentrations of PMs1 and PMs2 in LNCaP cells (A) and in PC3 cells (B) after incubation for 72 h. (n = 5).

PSMA is a validated molecular marker overexpressed by LNCaP cells [37], [38]. The cytotoxicity enhancing effect of PSMA ligand (SMLP) conjugated DTX-PMs were evaluated by in vitro cytotoxicity experiments using LNCaP and PC3 cells, respectively. The biocompatibility of PCL_12K_-mPEG_5K_ and PCL_12K_-PEG_5K_-SMLP was confirmed by incubating drug-free micelles composed of these two polymers at various concentrations with LNCaP cells and PC3 cells, respectively. The cell viability was not affected over a 72 h incubation period which confirmed the good biocompatibility of these polymers (Figure 7). The results also demonstrated that the cells cannot be interfered in the presence of ligands.

**Figure 8:**
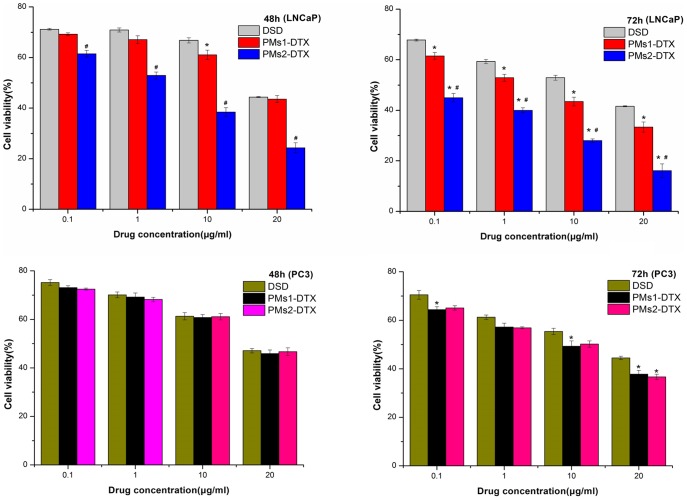
In vitro cytotoxicity determination of different concentrations of DSD, DTX-PMs1 and DTX-PMs2 in LNCaP cells and PC3 cells after incubation for 48 h, 72 h, respectively, using MTT assay. (*) significantly different from DSD; (#) significantly different from PMs1; (n = 5). Note: *P<0.01, ^#^P<0.01.

In this study, the DMSO solution of DTX (DSD) was used instead of Taxotere as a positive control because Tween 80 in Taxotere is cytotoxic and this may influence the results [39]. According to the results of the MTT assays (Figure 8), after a 48 h incubation, the cytotoxicity of PMs1 was nearly the same as DSD. However, PMs2 showed a significantly lower LNCaP cell viability at all concentrations. In PC3 cell lines, however, no significant difference was observed in cell viability among DSD, PMs1 and PMs2 (Figure 8). This indicated that ligand conjugation is beneficial in facilitating the cellular uptake of micelles: as PMs1 and PMs2 showed similar release profiles, the decreased cell viability could be ascribed to enhanced intracellular drug accumulation via receptor-mediated endocytosis. After 72 h incubation, both PMs1 and PMs2 showed significant differences from DSD in LNCaP cell line, and PMs2 showed the greatest cytotoxicity. Moreover, significant lower cell viabilities were observed in PC3 cell lines treated with either PMs1 or PMs2 than DSD at drug concentration of 20 µg/ml, which mean that inadequate cellular uptake of micelles could be compensated by increased incubation time and drug concentration. However, there were no significant differences between PMs1 and DSD after 48h incubation for LNCaP cells. This phenomenon confirmed the sustained drug release behavior of DTX from PMs1 *in vitro*.

The cell viability of PMs2 at 20 µg/ml was almost half that of PMs1 for LNCaP cells after either 48 h or 72 h incubation. These data demonstrated that DTX-PMs2 was less effective in enhancing cytotoxicity in PC3 cells, whereas it selectively inhibits proliferation of LNCaP cells. In the other words, these results suggest the high affinity of SMLP for PSMA could enhance the cytotoxicity in LNCaP cells. After 72 h incubation on LNCaP cells with the amount of DTX given at a fixed concentration of 20 µg/ml, the cell growth inhibition rate of DSD, PMs1 and PMs2 were 58.4%, 67.7% and 83.9%, respectively.

The IC50 for each sample is shown in Table 2. For LNCaP cells, the IC50 of DTX-PMs2 was much lower than that of DTX-PMs1 and DSD after 48 h or 72 h incubation. However, the IC50 of DTX-PMs1 was almost the same as that of DSD after 48 h incubation, but was 5-fold lower after 72 h incubation with LNCaP cells, this further confirmed the compensating effect in cytoxicity of micelles by prolonged incubation time. Although DTX-PMs2 (targeted) exhibited the highest cell-killing efficiency, the DTX-PMs1 also displayed an effect on LNCaP cells. Since the two micelles possessed similar release profiles, the differences in cytotoxicity were related to their different cell-entry ability which was reflected by the differences in affinities to PSMA.

**Table 2:** IC_50_ analysis of DSD, DTX-PMs1 and DTX-PMs2 on LNCaP and PC3 cells after 48 h, 72 h incubation, respectively (n = 5).

Incubation time (h)	IC_50_ values (µg/ml) of LNCaP cells IC_50_ values (µg/ml) of PC3 cells					
	DSD	DTX-PMs1	DTX-PMs2	DSD	DTX-PMs1	DTX-PMs2
48 h	14.59±2.11	13.48±1.03	0.87±0.27	19.31±3.42	18.65±3.07	19.04±2.81
72 h	7.36±1.51	1.35±0.54	0.02±0.008	11.67±1.98	3.11±0.81	3.02±0.76

### Cellular Uptake

**Figure 9:**
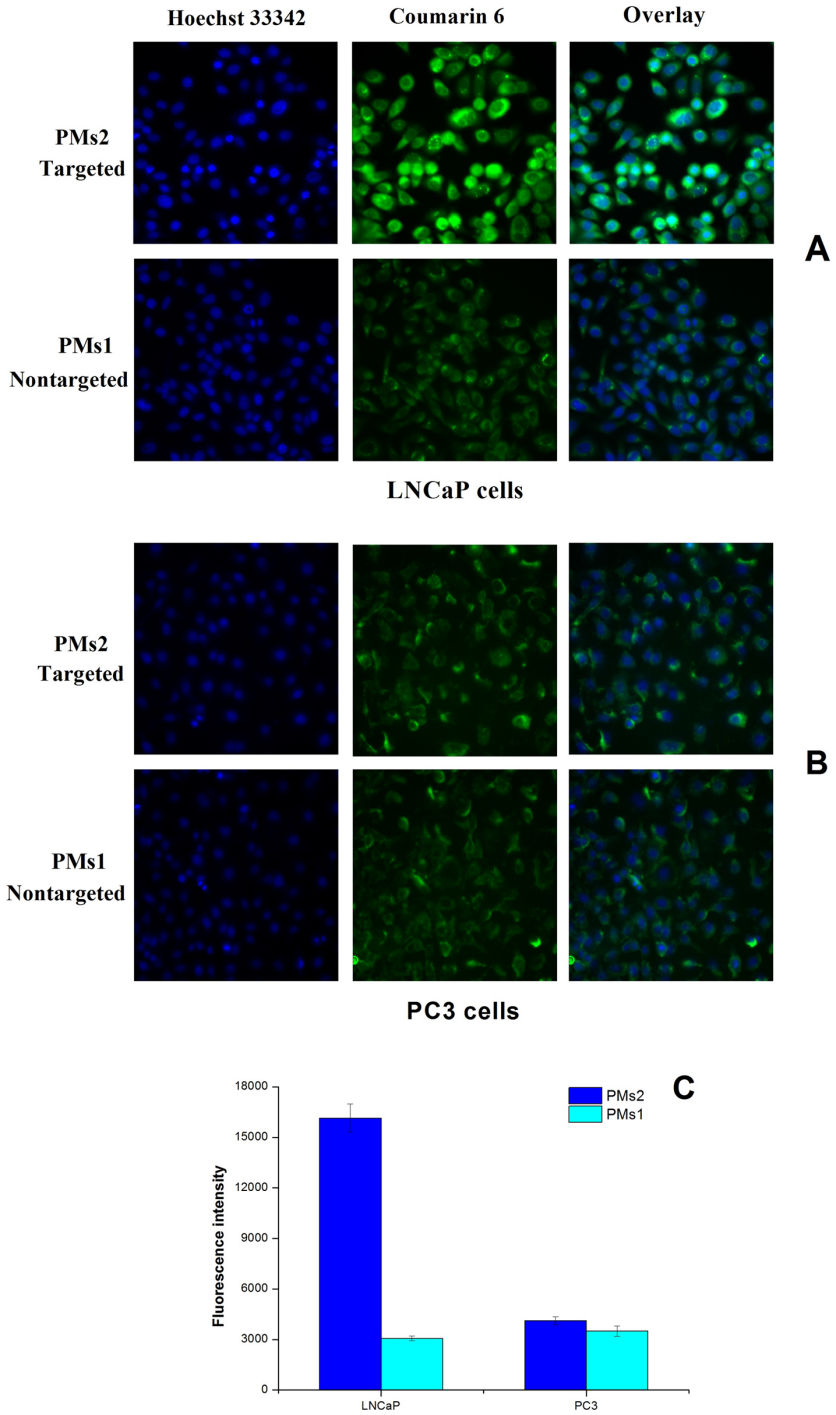
HCSS images of LNCaP (A) and PC3 cells (B) following a 4 h incubation at 37°C with coumarin 6-loaded PMs1 and PMs2, respectively. The cell nuclei were stained with Hoechst 33342 with the blue channel, the coumarin 6-loaded PMs are the green channel. The cellular uptake was visualized by overlaying images displayed by the nuclei channel and the PMs channel. The fluorescence intensity/cell graph of 100 µg/ml coumarin 6-loaded PMs1 and PMs2 with a concentration of 200 µg/ml after 4 h incubation with LNCaP cells and PC3 cells (C).

**Figure 10:**
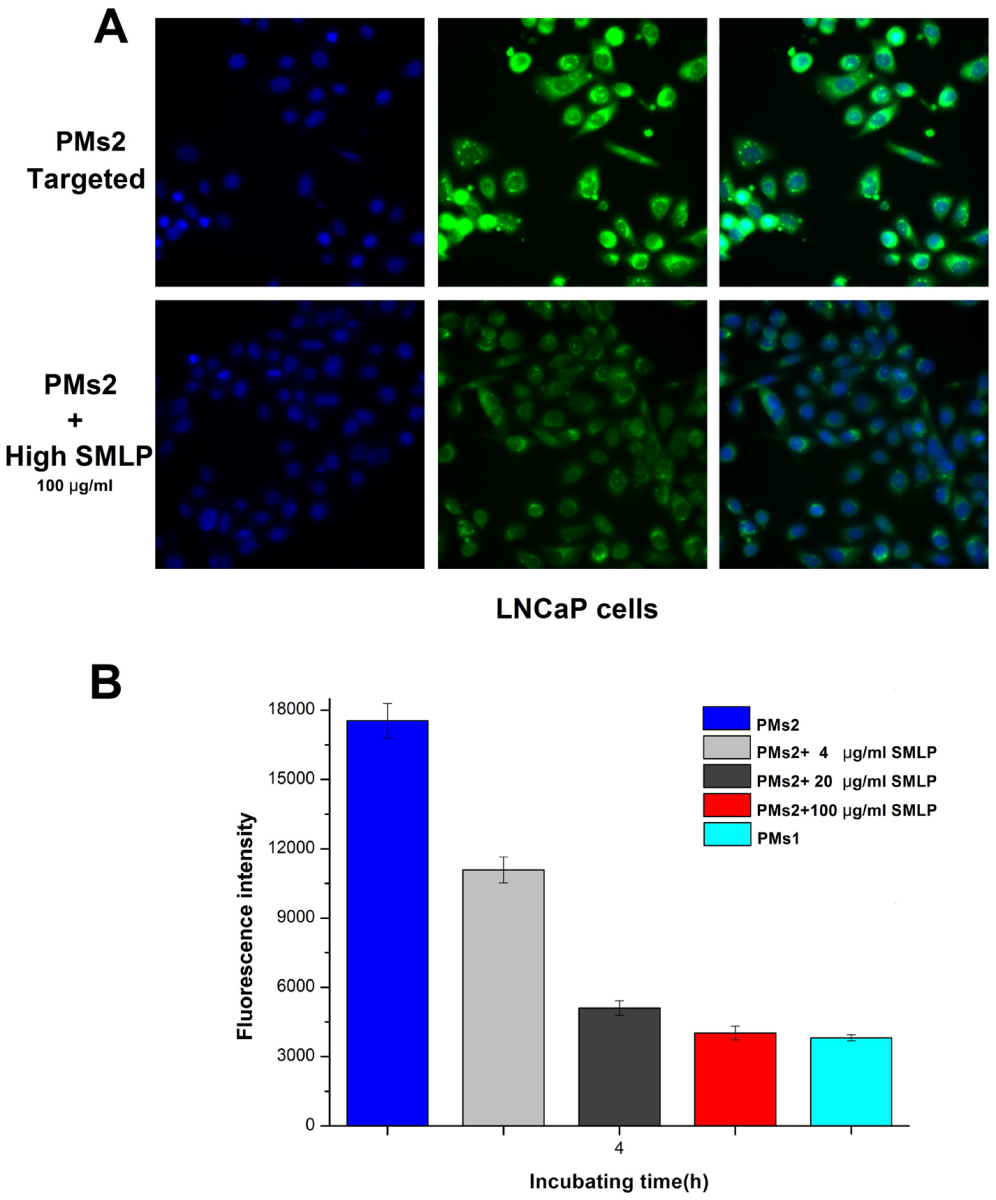
Competitive inhibition analysis of free SMLP (A) and fluorescence intensity/cell on LNCaP cells after 4 h incubation (B).

To study the effect of PSMA targeting ligand on the cellular uptake of the PMs, fluorescence microscopy was conducted on LNCaP cells with both targeted (PMs2) and non-targeted (PMs1) micelles labeled with coumarin-6. After 4 h incubation at 37°C, HCSS images of LNCaP cells were taken and shown in Figure 9A. The fluorescence intensity of cells incubated with targeted micelles (PMs2) was significantly higher than that of its non-targeted counterpart (PMs1), and the quantified fluorescence intensity was estimated to be 5-fold (Figure 9C). The capability of SMLP conjugation in enhancing cellular uptake could be reflected in cell viability assays. To further verify the role of SMLP in endocytosis, a ligand competing experiment was conducted. As shown in Figure 8B, addition of free ligands at various concentrations gradually decreased the uptake of PMs2, and the amount of endocytosed micelles reached a similar level to its non-targeted counterpart at high concentration of SMLP (100 µg/ml, Figure 10B), which indicated the presence of free SMLP in the medium inhibited endocytosis of PMs2 by binding to surface PSMA in a competitive manner against micelle-conjugated SMLP. To further verify the enhancement of ligand in mediating endocytosis, cellular uptake studies of both preparations with/without SMLP ligand were conducted in PC-3 cell line, which do not express the PSMA protein [40]. As shown in Figure 9 (B and C), targeted- and non-targeted micelles showed similar intracellular fluorescent intensity, which means conjugation of SMLP is a key factor in promoting cellular uptake of prepared micelles in PSMA expressing cells. Also the above results demonstrated that PMs2 was endocytosed into LNCaP cells via multiple routes: part of the micelles was taken up by LNCaP cells in a SMLP-mediated manner, while there were micelles entering cells through other pathways including caveolin-mediated endocytosis or clathrin- and caveolin-independent endocytosis [41] as the cellular uptake of micelles was not completely inhibited by SMLP addition. These results explained the higher cytotoxicity of the targeted micelles (PMs2) in MTT assays and indicated the benefit of PMs with a targeting ligand in prostate cancer therapy.

## Conclusions

In this study, a novel self-assembly of DTX-PEG-PCL-SMLP micelles targeting LNCaP cells was developed. With the same hydrophilic/hydrophobic block length ratio, a series of polymeric micelles with diameters less than 60 nm were prepared by dialysis. Stable non-targeted PMs and targeted PMs with constant drug-loading content were obtained by short-term stability assays. Reliable drug loading and sustained releasing behavior were obtained due to removal of the over-loaded drugs. The cyotoxicity experiments demonstrated the advantages in LNCaP cell inhibition with a significant difference of targeted DTX-PMs > non-targeted DTX-PMs > DSD. The fluorescence intensity of coumarin 6-loaded targeted PMs were 5-fold higher than that of non-targeted PMs. Combining the cellular uptake results of both targeted- and non-targeted micelles in LNCaP and PC3 cell lines, the critical role of SMLP conjugation in facilitating micelle uptake in PSMA positive cells was demonstrated. All of these results were ascribed to the ligand targeting of PSMA that guaranteed efficient uptake of micelles composed of DTX-PCL-PEG-SMLP that exhibited highest cytotoxicity on LNCaP cells. In summary, intracellular drug delivery is crucial for the anti-tumor efficacy of poorly-permeable drugs. As shown in this study, DTX-PCL-PEG-SMLP showed remarkable cytotoxicity compared with DMSO solution of DTX. DTX-PCL-mPEG also displayed higher cyotoxicity than DMSO solution of DTX due to enhanced intracellular accumulation via endocytosis of micelles. A more positive effect could be achieved by ligand conjugation that anchored micelles to tumor cells and facilitated cellular uptake. Further investigation into other properties of this drug delivery system, such as pharmacokinetics, *in vivo* antitumor activity and tissue distribution, are still required. Moreover, PCL-PEG-SMLP as a drug carrier is expected to be used in a number of ways for PCa therapy.
